# Low‐ dose Apatinib promotes vascular normalization and hypoxia reduction and sensitizes radiotherapy in lung cancer

**DOI:** 10.1002/cam4.5113

**Published:** 2022-09-06

**Authors:** Shanshan Jiang, Yue Zhou, Liqing Zou, Li Chu, Xiao Chu, Jianjiao Ni, Yida Li, Tiantian Guo, Xi Yang, Zhengfei Zhu

**Affiliations:** ^1^ Department of Radiation Oncology Fudan University Shanghai Cancer Center Shanghai China; ^2^ Department of Oncology, Shanghai Medical College Fudan University Shanghai China; ^3^ Institute of Thoracic Oncology, Fudan University Shanghai China

**Keywords:** apatinib, low‐dose, lung cancer, radiotherapy, vascular normalization

## Abstract

**Background and Purpose:**

Abnormal vascular network of tumor can create a hypoxic microenvironment, and reduce radiotherapy sensitivity. Normalization of tumor vasculature can be a new therapeutic strategy for sensitizing radiotherapy. This study aimed to explore the effect of apatinib on vascular normalization, as well as the syngeneic effect with radiotherapy on lung cancer.

**Materials and Methods:**

Lewis lung carcinoma (LLC) xenograft‐bearing female C57BL/6 mice were treated with different doses of apatinib (30, 60, and 120 mg/kg per day) and/or radiation therapy (8 Gy/1F) and then sacrificed to harvest tumor tissue for immunohistochemical test. Further ^18^F‐FMISO micro‐ PET in vivo explored the degree of hypoxia.

**Results:**

Immunohistochemistry of CD31 and alpha‐smooth muscle actin (α‐SMA) proved that low‐dose apatinib can normalize vasculature in tumor, especially on Day 10. Tissue staining of hypoxyprobe‐1 and ^18^F‐FMISO micro‐ PET in vivo showed that 60 mg/kg/day of apatinib significantly alleviates hypoxia. Moreover, this study further proved that low‐dose apatinib (60 mg/kg/day) can enhance the radio‐response of LLC xenograft mice.

**Conclusion:**

Our data suggested that low‐ dose apatinib can successfully induce a vascular normalization window and function as a radio‐ sensitizer in the lung cancer xenografts model.

## Graphical abstract

Low‐dose apatinib normalizes tumor blood vessels and significantly ameliorate intratumor hypoxic level in lung cancer. Meanwhile, low‐daose apatinib can sensitize radiotherapy in the xenograft mouse model, likely due to vascular normalization and hypoxia reduction.

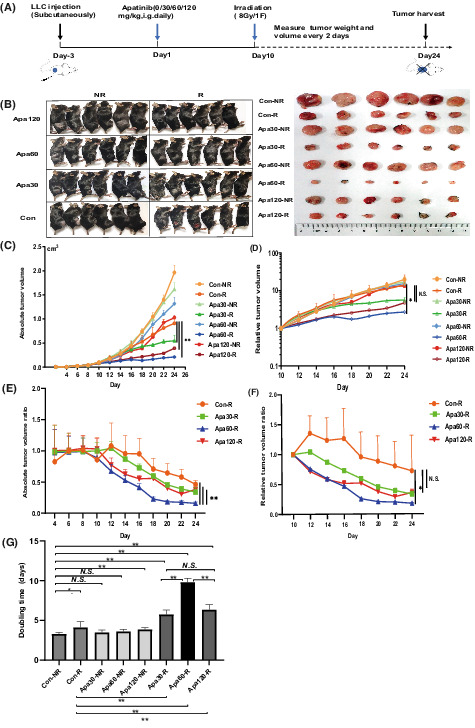

## 1 INTRODUCTION

Radiotherapy plays a pivotal role in non‐surgical treatment of lung cancer. However, radio‐resistance remains a serious issue because it leads to high recurrence rate and poor survival.^1^ Tumor hypoxia is an important mechanism of radiation resistance. Irregular distribution of vasculature and uneven blood flow can affect oxygen transport and impede tumor reoxygenation.^2^ Anti‐angiogenesis drugs were first developed to arrest tumor growth by choking off blood vessels to reduce the energy supply of cancer cells.^3^, ^4^ Accumulating data indicated that small doses of antiangiogenic drugs can make tumor vascular normalization,^5^, ^6^ and likely alleviate tumor hypoxia and potentiate effects of radiotherapy and chemotherapy.^7^, ^8^, ^9^, ^10^

Apatinib is an oral vascular endothelial growth factor 2 (VEGFR2) inhibitor, which is also the first small molecular anti‐angiogenesis agent applied in the third‐line therapy of gastric cancer by the China Food and Drug Administration in 2014. Moreover, promising anticancer activity has been proven in multiple malignant tumors, including hepatocellular cancer,^11^ colon cancer,^12^ lung cancer,^13^ and nasopharyngeal cancer.^14^ Several studies have explored the normalization of tumor vasculature of low‐ dose apatinib, which was later perceived as a sensitizer for chemotherapy^15^ and immunotherapy.^16^ Li et al.^17^ demonstrated that apatinib could inhibit the AKT and ERK signaling pathways of NSCLC, thereby improving its radiosensitivity. Liao et al.^18^ illustrated that apatinib can inhibit the radiation‐induced PI3K/AKT pathway and reduce the proliferation of hepatocellular carcinoma xenografts, showing its potential therapeutic effect. Moreover, apatinib combined with radiation therapy can improve the anticancer effect of nasopharyngeal carcinoma.^19^ However, the role of apatinib in sensitizing radiotherapy in the xenograft lung cancer model remains unverified. Therefore, the purpose of this study was to investigate the effect of apatinib on the normalization of blood vessels and the sensitivity of radiotherapy in vivo, so as to provide a reference for comprehensive treatment of lung cancer.

## 2 MATERIALS AND METHODS

### 2.1 Cell culture

Lewis lung carcinoma (LLC) cells were obtained from Chinese Academy of Sciences Library (Shanghai, China) and cultured at temperature of 37°C in a humidified incubator with a constant 5% CO_2_ in Dulbecco's modified Eagle medium (DMEM) (Life Technologies, Grand Island, NY), which contained 10% fetal bovine serum (FBS) (Life Technologies, Grand Island, NY),100 units/ml penicillin and 100 μg/ml streptomycin. These cells are cultured in certain numbers of generations and tested periodically to confirm that they are free of infection.

### 2.2 Xenograft mouse model

The experiments of mice gained Institutional Committee on Animal Care and Utilization of Fudan University Shanghai Cancer Research Center's (FUSCC) favor. C57BL/6 mice aged 6–8 weeks were obtained from Shanghai SLAC Experimental Animal Co., Ltd. (Shanghai, China). Animals have enough food and water in a sterile laboratory environment under alternating light (12 h light/12 h dark cycle), constant temperature (25°C) and suitable humidity (55%–10%). 1 × 10^6^ LLC cells resuspended in 150uL PBS were used to subcutaneously transplanted and established a subcutaneous tumor model. These tumor‐ bearing mice were examined in detail and their tumors were observed.

### 2.3 Treatment and measurement of tumor‐ bearing mouse

Apatinib was kindly provided by Jiangsu Hengrui Pharmaceuticals Co., Ltd., Shanghai, China, and dissolved in 0.5% (W/V) Carboxymethyl Cellulose ‐NA Solution (CMC‐ NA). Three days after tumor cells inoculation, different doses of apatinib were administered by oral gavage every day. A control group without apatinib treatment received similar volumes of 0.5% CMC‐ NA. The day starting the administration of apatinib was recorded as Day 1. As for radiotherapy, the tumor‐ bearing mouse was anesthetized with isoflurane (RWD Life Science, China) before radiotherapy at indicated day. Then, mice were positioned and received single dose of 8 Gy X‐ray with a 6 MeV electron beam (Siemens Primus‐Hi) based on previous studies.^17^, ^20^ The distance between radioactive source and tumor surface was 1 meter, and the radiation field was 2.5 × 2.5 cm^2^. The size of the tumor was quantitatively measured with a caliper every other day, and the result of tumor volume was defined as: (long axis) × (short axis) 2 × π/6.

### 2.4 Immunohistochemistry assay

After embedding in 10% neutral formalin‐fixed paraffin, tumor tissue sections were deparaffinized by hydration method and inhibited by H_2_O_2_. Tissue slides were stained with hematoxylin and eosin. In immunohistochemistry (IHC) for CD31 and α‐SMA, first use mAb CD31 (#77699, 1:200, Cell Signaling Technology) and α‐SMA (#19245, 1:600), then follow the manufacturer's instructions, stained with secondary antibody (HRP‐conjugated anti‐rabbit) which diluted in 1:100 for 1 h at room temperature, and finally counterstained with hematoxylin. Sections were analyzed under an A1 light microscope (Zeiss, Jena, Germany). Different slices were examined using Image‐J software (National Institutes of Health 1.8.0_112) to determine the threshold. Three fields of IHC sections were evaluated with a 20x microscope.

### 2.5 Hypoxia test

To study hypoxic areas in tumors, each mouse was given Hypoxyprobe‐1 (60 mg/kg, Hypox probeTM‐1 Supplement Kit, HyPoxyprobe, USA). One hour later, the tumor was removed and fixed with 4% paraformaldehyde. Tumor tissues were then sectioned and stained for monoclonal antibody (Mab1) and F(ab’)2 antibody‐conjugated HRP secondary antibody, following the manufacturer's recommendations. Sections were then re‐stained with hematoxylin. Quantification was performed by five random views of each tumor over a 5x‐field of view using ImageJ (NIH 1.8.0_112).

### 2.6 ^18^F‐ FMISO‐ based micro‐ PET

Tumor‐bearing mice were scanned for hypoxia in vivo using PET/CT (PET/CT) (Inveon, Siemens, Micro PET research center of FUSCC) based on ^18^F‐fluoromiisonidazole (^18^F‐FMISO). 5.55 MBq (150 μg) of ^18^F‐FMISO were administered by tail vein injection. For best contrast, PET images were performed after ^18^F‐FMISO for 90 min. One‐percent isoflurane was used to anesthetize the mice prior to scanning and maintained during scanning. Inveon Acquisition Workplace (version 2.0, Siemens Preclinical Solutions) was used to reconstruct PET images. The uptake of ^18^F‐FMISO in tumors was evaluated semi‐quantitatively, and the maximum normalized uptake values (SUVmax) of different mouse tumors were obtained to evaluate the maximal radiation concentration within region of interest (ROI).

### 2.7 Statistical analysis

Data are presented as numerical values and percentages, with mean‐standard deviation (*SD*). The analysis between two groups and more than three groups used t‐test and the analysis of variance (ANOVA), respectively. All statistical analyses were performed by the SPSS 25.0 (IBM Inc, USA). Statistically significance was defined as *P* < 0.05.

## 3 RESULTS

### 3.1 Low‐ dose apatinib induced a vascular normalization window in vivo

**FIGURE 1:**
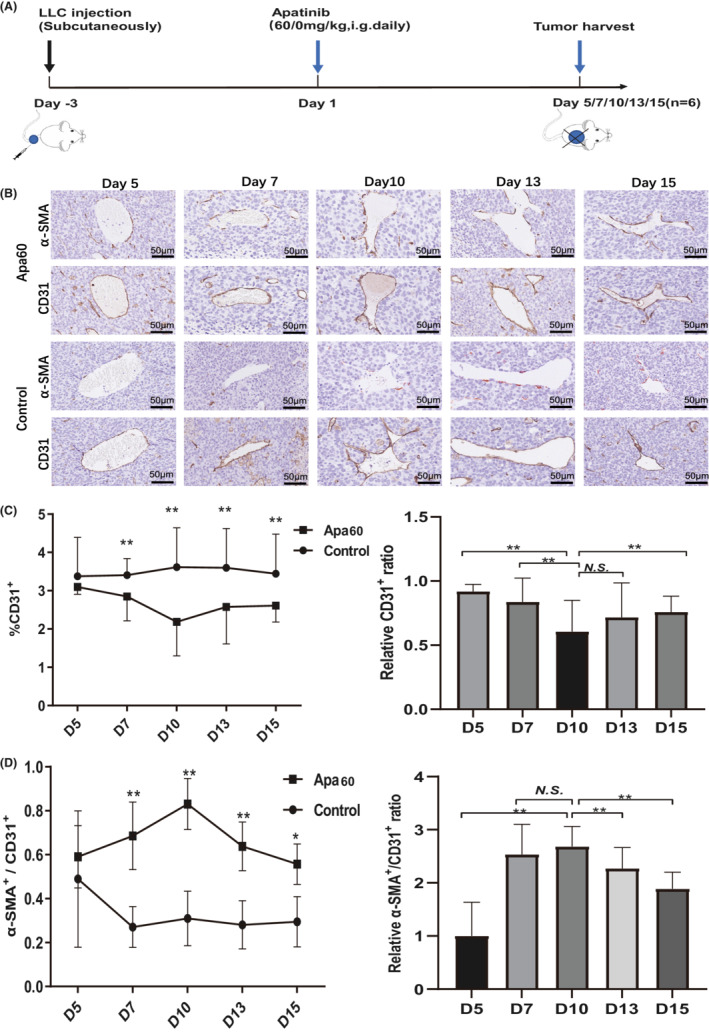
Low‐ dose apatinib induced a vascular normalization window in vivo. Sixty tumor bearing mouse were divided (30 mice per group) and treated with low‐ dose apatinib (60 mg/kg/day, Apa60 group) or CMC (control group). Mice (6 per group) were sacrificed and tumor tissues were obtained on Day 5, 7, 10, 13, and 15, respectively. (A) Treatment schedule of tumor‐bearing mouse; (B) Representative images of CD31 and α‐SMA staining; (C) Representative changes of the ratio of CD31+ area and relative CD31+ ratio; (D) Dynamic changes of the ratio of α‐SMA+/CD31+ and relative α‐SMA+/ CD31+ ratio. Data was presented as mean, and error bars represented the *SD*. *P < 0.05; **P < 0.01; N.S., no significance.

Previous study has shown that VEGFR2 blockage normalizes blood vessels within a time window, thereby reducing hypoxia.^21^ Thus, we aimed to examine the time window of vascular normalization after apatinib treatment. According to previous studies, low‐ dose anti‐ angiogenic agents can improve tumor microenvironment.^16^, ^22^ Therefore, 60 mice with LLC xenograft were divided (30 mice per group) and treated with low‐ dose apatinib (60 mg/kg/day, Apa60 group) or CMC (control group). Mice were sacrificed (6 in each group) and tumor tissue was harvested on Day 5, 7, 10, 13, and 15, respectively. Treatment protocol was shown in Figure 1A. Representative images of IHC results were shown in Figure 1B. CD31 staining demonstrated that 60 mg/kg/day of apatinib prominently reduced the vascularity of the LLC xenografts compared with control group, especially on Day 10 (*P* < 0.01) (Figure 1C). Also, the ratio and relative ratio of α‐SMA to CD31 positive area (α‐SMA+/CD31+), which reflect the coverage rate of mature pericytes, revealed that Apa60 group strikingly increased mature pericytes coverage than those in the control group (*P* < 0.05), especially on Day 10 (Figure 1D).

### 3.2 Low‐ dose apatinib alleviated hypoxia in vivo

**FIGURE 2:**
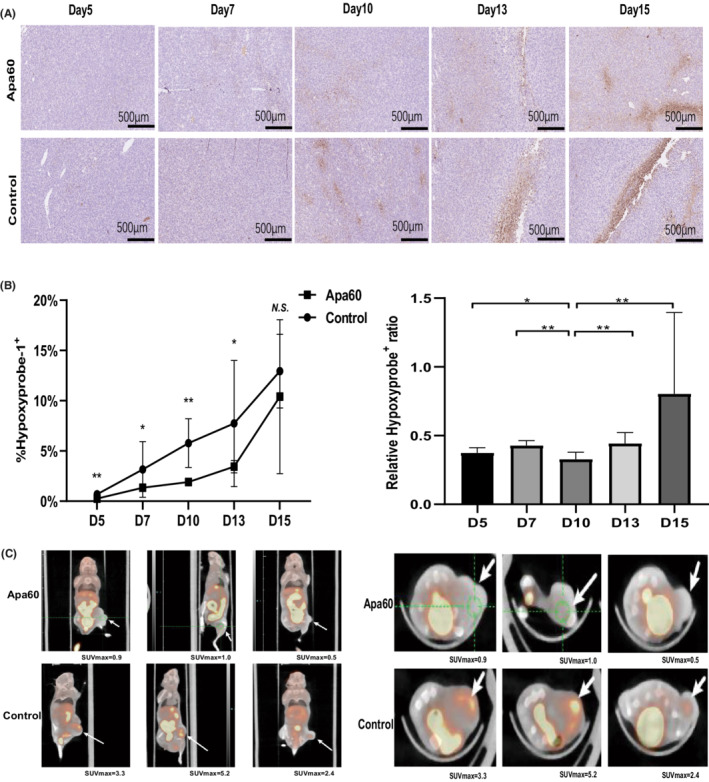
Low‐ dose apatinib alleviated hypoxia in vivo. Sixty tumor bearing mouse were divided (30 mice per group) and treated with low‐ dose apatinib (60 mg/kg/day, Apa60 group) or CMC (control group). Tumor tissues were harvested and stained with anti‐ Hypoxyprobe‐1 antibodies on Day5, Day7, Day10, Day13, and Day15 after apatinib initiation (*n* = 5 per group at different time). (A) Representative images of Hypoxyprobe‐1 staining; (B) Dynamic changes of the ratio of Hypoxyprobe‐1+ aera and relative Hypoxyprobe‐1+ ratio; (C) Sagittal section and coronal section of ^18^F‐FMISO based micro‐ PET images of LLC xenograft (*n* = 3 per group on Day 10). White arrow represents the location of the tumor. SUVmax, the maximum standardized uptake value. Data was presented as mean, and error bars represented the *SD*. *P < 0.05; **P < 0.01; N.S., no significance.

The hypoxic area of tumor tissues was verified by hypoxyprobe‐1 staining (Figure 2A). As presented in Figure 2B, hypoxyprobe‐1 staining revealed that Apa60 group significantly improved tumor hypoxia on Day 10 compared with control group (*P* < 0.01), and then the difference gradually declined with time elapse. Based on above results, the potential effect of alleviation hypoxia in vivo was further investigated by ^18^F‐FMISO based micro‐PET on Day 10 (*n* = 3 per group). Results demonstrated that the SUVmax value of Apa60 group (0.80 ± 0.22) was significantly lower than control group (3.63 ± 1.17) (*P* = 0.03), indicating that the hypoxia level in tumor with apatinib treatment on Day 10 was remarkably alleviated (Table 1, Figure 2C). However, no significant difference was observed in body weight and tumor volume of mice between two groups (*P* > 0.05) (Table 1). These findings suggested low‐ dose apatinib was able to improve vascular normalization and hypoxia, especially around Day 10. Hence, Day 10 was chosen as vascular normalization window for further experiment.

**TABLE 1:** Comparison of SUVmax, body weight and tumor volume between Con and Apa60 group

	Con	Apa60	*P* value
SUVmax	3.63 ± 1.17	0.80 ± 0.22	0.03
Body weight (g)	17.65 ± 0.39	17.47 ± 0.43	0.68
Tumor Volume (cm^3^)	0.09 ± 0.02	0.08 ± 0.01	0.63

Abbreviations: Apa60, apatinib 60 mg/kg/day group; Con, control group; SUVmax, the maximum standardized uptake value; *n* = 3 per group.

### 3.3 Different doses of apatinib in vascular normalization and hypoxia reduction

**FIGURE 3:**
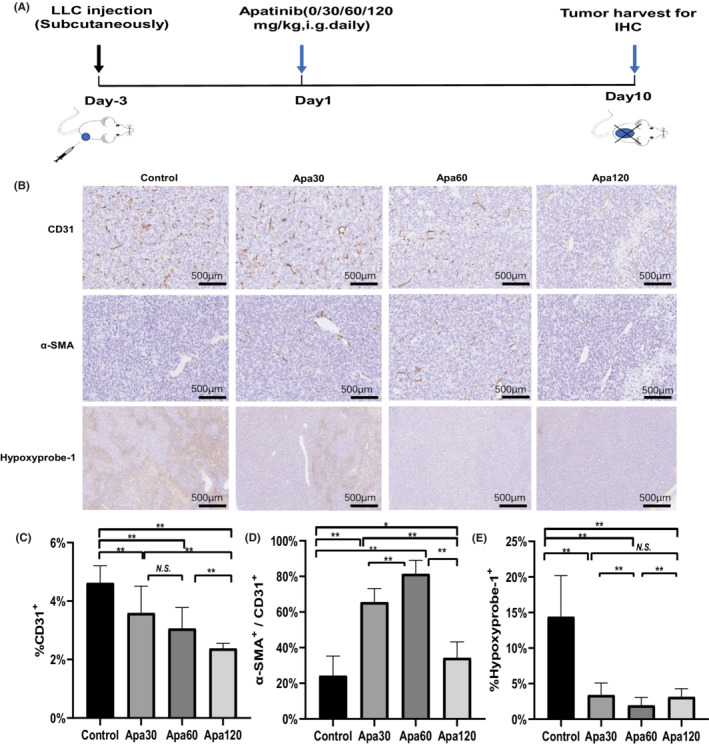
Different doses of apatinib in vascular normalization and hypoxia reduction. LLC xenograft‐ bearing mice were randomly divided into 4 groups (control, Apa30, Apa60, Apa120, *n* = 6 per group). Tumor tissues were harvested and stained with anti‐CD31, anti‐α‐SMA and anti‐Hypoxyprobe antibodies on Day10 after apatinib initiation. (A) Treatment schedule of tumor‐ bearing mouse; (B) Representative images of CD31, α‐SMA and Hypoxyprobe‐1 staining; (C) The ratio of CD31+ aera; (D) The ratio of α‐SMA+/CD31+ aera; (E) The ratio of Hypoxyprobe‐1+ aera. Data was presented as mean, and error bars represented the *SD*. *P < 0.05; **P < 0.01; N.S., no significance.

Previous conclusions were controversial whether high‐ dose apatinib was able to show more effect on vascular normalization and tumor suppression.^16^, ^18^, ^23^ We then titrated the dose of apatinib to explore the optimal dose of apatinib for vascular normalization. Twenty‐four tumor‐bearing mice were divided into 4 groups randomly for comparison, and received 30 mg/kg (Apa30 group), 60 mg/kg (Apa60 group), 120 mg/kg (Apa120 group) apatinib and CMC (control group) per day, respectively. On Day 10, tumor tissues were harvested and processed for IHC staining with antibody against CD31, α‐SMA and Hypoxyprobe‐1 (Figure 3A). Representative images of IHC of different groups were illustrated in Figure 3B. The results of micro‐vessel density (measured by CD31+) and pericyte coverage (measured by α‐SMA+/CD31+) showed that the vascular normalization was better in apatinib group than in control group, with the best effect in Apa60 group (Figure 3C,D). In addition, hypoxyprobe‐1 staining showed that 60 mg/kg/day of apatinib remarkably alleviates hypoxia compared with other groups (*P* < 0.001) (Figure 3E). Overall, the dosage of apatinib with 60 mg/kg/day has the better ability of vascularization and reduction of hypoxia. These results showed that apatinib was able to promote vascular normalization, while low‐ dose apatinib exerted a more promising effect.

### 3.4 Apatinib sensitizing radiotherapy in vivo

**FIGURE 4:**
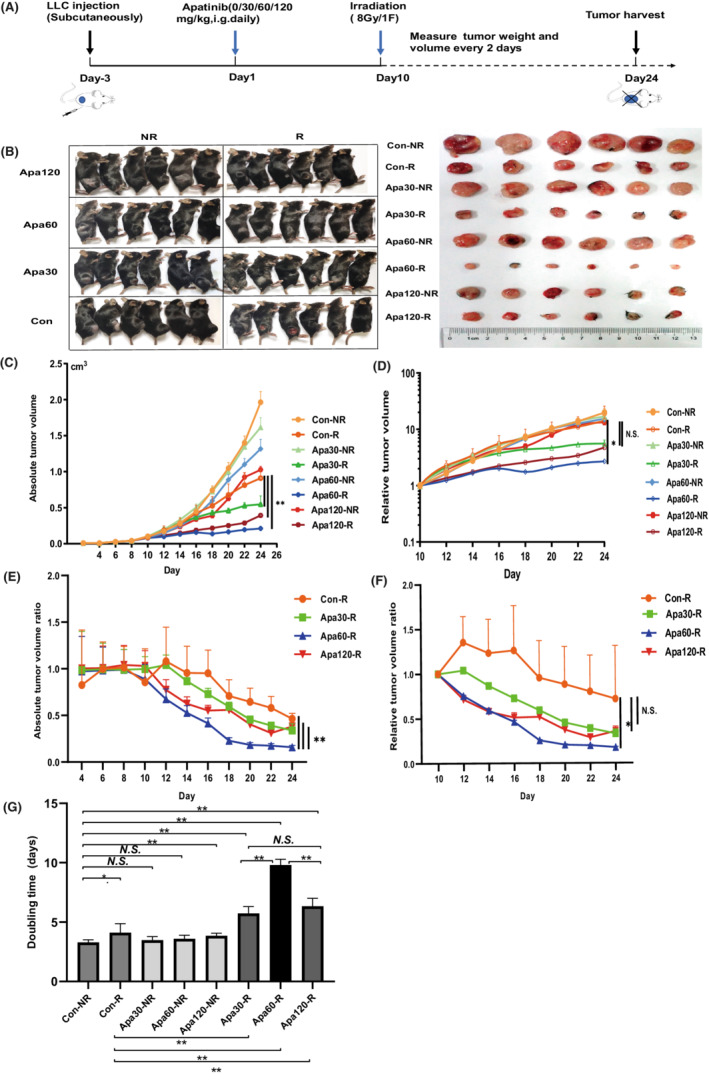
Apatinib sensitizing radiotherapy in vivo. LLC xenograft‐ bearing mice were evenly divided into 4 groups with radiotherapy (8Gy/1F) (Con‐R, Apa30‐R, Apa60‐R, and Apa120‐R, *n* = 6 per group) and four groups without radiotherapy (Con‐NR, Apa30‐NR, Apa60‐NR and Apa120‐NR, *n* = 6 per group). Con, control group; R, radiotherapy; NR, no radiotherapy. (A) Treatment schedule of tumor‐ bearing mouse; (B) Images of tumor‐ bearing mouse and tumor volume on Day 24; C) Measurement of absolute tumor volumes; (D) Measurement of relative tumor volumes; (E) Measurement of absolute tumor volume ratio; (F) Measurement of relative tumor volume ratio; (G) Tumor doubling times of different groups. Data was indicated as mean, and error bars represented the *SD*. *P < 0.05; **P < 0.01; N.S., no significance.

To explore whether this effect of apatinib can be transformed into sensitizing effect on radiotherapy, tumor‐bearing mouse were treated with 0/30/60/120 mg/kg/day apatinib and/or 8Gy irradiation (Figure 4A). Images of LLC xenograft‐bearing mouse and tumor size were shown in Figure 4B. Notably, three combined treatment groups (Apa30‐R, Apa60‐R, and Apa120‐R, ‘R' is the abbreviation of radiotherapy) were more effective on delaying tumor growth than either apatinib or radiotherapy alone. Besides, among combined treatment groups, Apa60‐R had best efficacy on delaying tumor growth both on absolute and relative tumor volume compared with Con‐R group (*P* < 0.01, *P* < 0.05, respectively) (Figure 4C,D). Moreover, standardized absolute tumor volume and relative tumor volume also elucidated that 60 mg/kg/day of apatinib combined with radiotherapy showed better therapeutic effect on shrinking tumor volume (*P* < 0.01, *P* < 0.05, respectively) (Figure 4E,F). As shown in Figure 4G, the doubling times of all combined group were significantly longer than Con‐NR and Con‐R (for all, *P* < 0.01), suggesting the radio‐sensitizing effect of apatinib. Among the three combined groups, Apa60‐R (9.80 ± 0.45 days) showed prominently longer doubling time compared to Apa30‐R (5.73 ± 0.53 days) and Apa120‐R (6.33 ± 0.61 days) (*P* < 0.01). Furthermore, Apa60‐R significantly enhanced the response to radiotherapy compared to Apa30‐R and Apa120‐R, with the enhancement ratio of 7.64 in Apa60‐R group, 3.07 in Apa120‐R group and 2.77 in Apa30‐R group, respectively (Table 2). It is suggested that the combined use of radiotherapy and low‐ dose apatinib has a synergistic effect on lung cancer.

**TABLE 2:** The growth of tumor in different groups of xenograft mouse

Treatment	Doubling time (days)	Absolute growth delay (days)	Normalized growth delay (days)	Enhancement ratio
Con‐NR	3.29 ± 0.20	/	/	/
Con‐R	4.10 ± 0.70	0.81(4.10–3.29)	/	/
Apa30‐NR	3.48 ± 0.27	0.19(3.48–3.29)	/	/
Apa30‐R	5.73 ± 0.53	2.44(5.73–3.29)	2.25(2.44–0.19)	2.77(2.25/0.81)
Apa60‐NR	3.60 ± 0.27	0.31(3.60–3.29)	/	/
Apa60‐R	9.80 ± 0.45	6.51(9.80–3.29)	6.20(6.51–0.31)	7.65(6.20/0.81)
Apa120‐NR	3.84 ± 0.13	0.55(3.84–3.29)	/	/
Apa120‐R	6.33 ± 0.61	3.04(6.33–3.29)	2.49(3.04–0.55)	3.07(2.49/0.81)

Abbreviations: Apa30, apatinib 30 mg/kg/day; Apa60, apatinib 60 mg/kg/day; Apa120, apatinib 120 mg/kg/day; Con, control group; R, radiotherapy; NR, no radiotherapy; *n* = 6 per group.

*Note*: The data are presented as mean ± standard deviation. Absolute growth delay was calculated as “Each group doubling time‐Con‐NR doubling time”; Normalized growth delay was calculated as “Apa‐R absolute growth delay‐ Apa‐NR absolute growth delay”; Enhancement ratio was calculated as “Normalized growth delay/Con‐R absolute growth delay”.

## 4 DISCUSSION

Radiotherapy is a well‐ established therapeutic treatment in NSCLC. However, despite the continuous improvement of radiotherapy technology, the local control of NSCLC is still unsatisfactory, and the local recurrence rate of stage III NSCLC after radiotherapy alone can be as high as 60%–80%.^24^ Currently, radiotherapy resistance and local recurrence are main problems that must be considered in lung cancer radiotherapy.^25^ Anti‐angiogenesis drugs have obvious inhibitory effect on tumor vascular density, and the combined application of some marketed angiogenesis drugs and radiotherapy shows their synergistic effect.^20^, ^21^

Apatinib is an oral small‐molecule drug that inhibits VEGFR2 and has anti‐angiogenesis effects.^26^ For example, apatinib can promote apoptosis and autophagy and then suppress tumor proliferation in thyroid cancer,^27^, ^28^ colorectal cancer,^29^, ^30^ lung cancer,^31^, ^32^ and esophageal carcinoma.^33^ In addition, this anti‐ angiogenic agent exhibits an explicit effect on vascular normalization.^15^ Apatinib may also block VEGF and PI3K/AKT pathways and further inhibit tumor cell migration, invasion and angiogenesis.^34^ Moreover, apatinib has been applied to several clinical trials for the treatment of cancers. For example, a clinical study enrolled patients with terminal cervical cancer revealed that apatinib plus immunotherapy indicated promising antitumor activity and manageable toxicities.^35^ Another phase II clinical trial included patients with advanced liver cancer who were naïve or unable to receive first‐line targeted therapy. The results show that the combination of apatinib and Camrelizumab has good effect and controllable safety.^36^ The apatinib combination treatment presented superior survival in advanced EGFR‐mutant NSCLC when administered as first‐line therapy.^37^

Vascular normalization plays an important role in radio‐ sensitization, and a previous study demonstrated that the anti‐VEGFR2 therapy attenuates intra‐tumoral hypoxia by modulating tumor vasculature.^38^ The vascular normalization window is a period of time in which tumor blood flow and oxygenation transiently increase, can properly deliver chemotherapeutic agents, and enhance radiation therapy to potentiate anti‐tumor efficacy.^7^ Studies have revealed that apatinib improves the tumor vascular structure and thus induces vascular normalization with the advent of VEGFR2 blockades.^15^, ^39^ We firstly established the LLC xenograft‐bearing mouse model and demonstrated that 60 mg/kg/day of apatinib significantly promotes tumor vascular normalization and ameliorates hypoxia 10 days after the treatment. The results were then confirmed via ^18^F‐ FMISO based micro‐ PET examination in vivo further. Similar to previous study,^15^ apatinib improved the morphology of tumor blood vessels and reduced the spaces between endothelial cells within the normalization window (7 to 10 days after treatment). Our study demonstrated that apatinib significantly promotes tumor vascular normalization and hypoxia amelioration on Day 10 compared with other time points (Day 5, 7, 13 and 15 after apatinib treatment). Notably, vascular structure and tumor vessel function are transiently normalized and degenerated after anti‐angiogenic therapy. Therefore, selecting an appropriate time for conducting treatment combinations after anti‐angiogenic agent administration is important to reach the optimal therapeutic effect. As expected, the present study validated that low‐dose apatinib weakened hypoxia in tumor. Compared with the study of Liu et al.,^39^ which determined that apatinib can normalize tumor vascular on the dose and timing dependent manner (250 mg per day for 5 days) in NSCLC, our work obtained similar results of vascular normalization in the LLC xenograft mouse model despite the weakened the dose of apatinib (60 mg/kg/day). Similarly, a previous study reported that the low‐ dose apatinib (60 mg/kg/day) combined with immunotherapy alleviates hypoxia on Day 7 and increases anti‐ tumor efficacy in lung cancer.^16^ A dose escalation and safety study of phase Ia in hepatocellular carcinoma revealed that a relatively low‐ dose apatinib (125 mg/day) can reduce protocol‐ defined dose‐ limiting toxicity compared with cohorts of 250 and 500 mg/day.^40^ Collectively, these findings suggested that a suitable dose of apatinib can meet optimal treatment efficacy within a certain period of time.

On the basis of this theory, we further explored whether this amelioration in hypoxia can be transformed into a sensitizing effect on radiotherapy through the low‐ dose apatinib at a certain time window. The xenograft model was constructed and treated with different doses of apatinib and radiotherapy. Experiments in vivo subsequently validated that tumor‐bearing mouse treated with 60 mg/kg/day of apatinib combined with radiotherapy can significantly delay tumor growth and size while doubling the time at 10 days after treatment. The effect is superior to that of the administration of apatinib or radiotherapy alone. This finding demonstrated that low‐ dose apatinib can potentiate irradiation within the normalization window. The underlying mechanism of the degree of sensitization to radiotherapy is possibly related to the improvement of vessel normalization and alleviation of hypoxia. Similar to the results of previous studies, significant improvement of tumor blood infusion and oxygenation via vascular normalization can potentiate radiotherapy.^7^, ^8^, ^41^, ^42^ Although our data illustrated that low‐ dose apatinib can significantly enhance the effectiveness of radiotherapy at a certain time window, our work still needs further investigation in clinical trials. Notably, the sensitization of radiotherapy by apatinib may also be achieved through other mechanisms. For example, one study showed that apatinib can enhance PI3K/AKT pathway by inhibiting radiation‐induced DNA double‐strand breaks.^18^ In addition, apatinib can improve the radiosensitivity of NSCLC, and its mechanism may be related to AKT and ERK signaling pathways.^17^ Combination treatment of apatinib and radiotherapy on tumor may achieve systemic control by altering the tumor microenvironment.^23^ Therefore, apatinib is expected to become a clinical radiotherapy synergist and a new small molecule radiosensitizer.

This is the first study of combining low‐ dose apatinib and irradiation therapy applied in lung cancer in vivo. Notwithstanding our research found a potential effect of apatinib normalizes vascularization to sensitizing radiotherapy, the underlying mechanism of vascularization should be further uncovered in future. We referred to previous studies^41^, ^43^ and ruled out large doses of radiotherapy and fractional radiotherapy in the design. Therefore, the optimal combination of apatinib with conventional radiotherapy warrants further investigation. Our present report is only limited to the LLC subcutaneous tumor model. In future, additional studies with eligible lung cancer patients are merited to explore the efficacy and safety of apatinib plus radiotherapy.

## 5 CONCLUSION

Our data suggest that apatinib normalizes tumor blood vessels at lower dose and significantly ameliorate intratumor hypoxic level in lung cancer. Low‐ dose apatinib can sensitize radiotherapy in the xenograft mouse model, likely due to vascular normalization and hypoxia reduction. The results of our study can provide a reference for the clinical treatment of lung cancer. Issues on the optimal combination strategy in lung cancer patients and potential mechanisms of vessel normalization can be the focus of further investigations.

## AUTHOR CONTRIBUTIONS

Conceptualization: Shanshan Jiang, Xi Yang and Zhengfei Zhu. Data curation: Yue Zhou, Liqing Zou, Xi Yang, Li Chu, Xiao Chu, Jianjiao Ni, Yida Li, Tiantian Guo. Formal analysis: Yue Zhou and Tiantian Guo. Methodology: Xi Yang and Zhengfei Zhu. Funding acquisition: Zhengfei Zhu. Writing‐ original draft: Shanshan Jiang, Liqing Zou and Xi Yang. Writing‐ review and editing: Yue Zhou and Xi Yang. Study supervision: Xi Yang and Zhengfei Zhu.

## CONFLICT OF INTEREST

The authors declare no conflict of interest.

## ETHICS STATEMENT

This study was approved by the Institutional Committee for Animal Care and Use, Fudan University Shanghai Cancer Center, and were performed in accordance with the institutional guidelines.

## Data Availability

The datasets used and/or analyzed during the current study are available from the corresponding author on reasonable request.

## References

[1] Baker S , Dahele M , Lagerwaard FJ , Senan S . A critical review of recent developments in radiotherapy for non‐small cell lung cancer. Radiat Oncol. 2016;11:115. 27600665 10.1186/s13014-016-0693-8 PMC5012092

[2] Shannon AM , Bouchier‐Hayes DJ , Condron CM , Toomey D . Tumour hypoxia, chemotherapeutic resistance and hypoxia‐related therapies. Cancer Treat Rev. 2003;29:297‐307. 12927570 10.1016/s0305-7372(03)00003-3

[3] Teleanu RI , Chircov C , Grumezescu AM , Teleanu DM . Tumor angiogenesis and anti‐angiogenic strategies for cancer treatment. J Clin Med. 2019;9(1):84. 31905724 10.3390/jcm9010084 PMC7020037

[4] Folkman J . Tumor angiogenesis: therapeutic implications. N Engl J Med. 1971;285:1182‐1186. 4938153 10.1056/NEJM197111182852108

[5] Chen Q , Xu L , Chen J , et al. Tumor vasculature normalization by orally fed erlotinib to modulate the tumor microenvironment for enhanced cancer nanomedicine and immunotherapy. Biomaterials. 2017;148:69‐80. 28968536 10.1016/j.biomaterials.2017.09.021

[6] Huang G , Chen L . Recombinant human endostatin improves anti‐tumor efficacy of paclitaxel by normalizing tumor vasculature in Lewis lung carcinoma. J Cancer Res Clin Oncol. 2010;136:1201‐1211. 20130910 10.1007/s00432-010-0770-6 PMC11827984

[7] Jain RK . Normalizing tumor vasculature with anti‐angiogenic therapy: a new paradigm for combination therapy. Nat Med. 2001;7:987‐989. 11533692 10.1038/nm0901-987

[8] Myers AL , Williams RF , Ng CY , Hartwich JE , Davidoff AM . Bevacizumab‐induced tumor vessel remodeling in rhabdomyosarcoma xenografts increases the effectiveness of adjuvant ionizing radiation. J Pediatr Surg. 2010;45:1080‐1085. 20620299 10.1016/j.jpedsurg.2010.02.068 PMC2904306

[9] Tolaney SM , Boucher Y , Duda DG , et al. Role of vascular density and normalization in response to neoadjuvant bevacizumab and chemotherapy in breast cancer patients. Proc Natl Acad Sci U S A. 2015;112:14325‐14330. 26578779 10.1073/pnas.1518808112 PMC4655544

[10] McGee MC , Hamner JB , Williams RF , et al. Improved intratumoral oxygenation through vascular normalization increases glioma sensitivity to ionizing radiation. Int J Radiat Oncol Biol Phys. 2010;76:1537‐1545. 20338480 10.1016/j.ijrobp.2009.12.010 PMC2846307

[11] Qin S , Li Q , Gu S , et al. Apatinib as second‐line or later therapy in patients with advanced hepatocellular carcinoma (AHELP): a multicentre, double‐blind, randomised, placebo‐controlled, phase 3 trial. Lancet Gastroenterol Hepatol. 2021;6:559‐568. 33971141 10.1016/S2468-1253(21)00109-6

[12] Song Z , Lin Y , Zhang X , et al. Cyclic RGD peptide‐modified liposomal drug delivery system for targeted oral apatinib administration: enhanced cellular uptake and improved therapeutic effects. Int J Nanomedicine. 2017;12:1941‐1958. 28331317 10.2147/IJN.S125573 PMC5354530

[13] Li F , Zhu T , Cao B , Wang J , Liang L . Apatinib enhances antitumour activity of EGFR‐TKIs in non‐small cell lung cancer with EGFR‐TKI resistance. Eur J Cancer. 2017;84:184‐192. 28822888 10.1016/j.ejca.2017.07.037

[14] Peng QX , Han YW , Zhang YL , et al. Apatinib inhibits VEGFR‐2 and angiogenesis in an in vivo murine model of nasopharyngeal carcinoma. Oncotarget. 2017;8:52813‐52822. 28881773 10.18632/oncotarget.17264 PMC5581072

[15] Zhou K , Zhang JW , Wang QZ , et al. Apatinib, a selective VEGFR2 inhibitor, improves the delivery of chemotherapeutic agents to tumors by normalizing tumor vessels in LoVo colon cancer xenograft mice. Acta Pharmacol Sin. 2019;40:556‐562. 29977004 10.1038/s41401-018-0058-y PMC6461765

[16] Zhao S , Ren S , Jiang T , et al. Low‐dose Apatinib optimizes tumor microenvironment and potentiates antitumor effect of PD‐1/PD‐L1 blockade in lung cancer. Cancer Immunol res. 2019;7:630‐643. 30755403 10.1158/2326-6066.CIR-17-0640

[17] Li L , Li Y , Zou H . A novel role for apatinib in enhancing radiosensitivity in non‐small cell lung cancer cells by suppressing the AKT and ERK pathways. Peer J. 2021;9:e12356. 34760374 10.7717/peerj.12356 PMC8557687

[18] Liao J , Jin H , Li S , et al. Apatinib potentiates irradiation effect via suppressing PI3K/AKT signaling pathway in hepatocellular carcinoma. J Exp Clin Cancer res. 2019;38:454. 31694662 10.1186/s13046-019-1419-1 PMC6836669

[19] Liu S , Wu F , Zhang Y , et al. Apatinib combined with radiotherapy enhances antitumor effects in an in vivo nasopharyngeal carcinoma model. Cancer Control. 2020;27:1148357817. 10.1177/1073274820922553 PMC7235534 32420748

[20] Hu C , Zhu P , Xia Y , Hui K , Wang M , Jiang X . Role of the NRP‐1‐mediated VEGFR2‐independent pathway on radiation sensitivity of non‐small cell lung cancer cells. J Cancer res Clin Oncol. 2018;144:1329‐1337. 29777301 10.1007/s00432-018-2667-8 PMC11813453

[21] Winkler F , Kozin SV , Tong RT , et al. Kinetics of vascular normalization by VEGFR2 blockade governs brain tumor response to radiation: role of oxygenation, angiopoietin‐1, and matrix metalloproteinases. Cancer Cell. 2004;6:553‐563. 15607960 10.1016/j.ccr.2004.10.011

[22] Carmeliet P , Jain RK . Principles and mechanisms of vessel normalization for cancer and other angiogenic diseases. Nat Rev Drug Discov. 2011;10:417‐427. 21629292 10.1038/nrd3455

[23] Liang LJ , Hu CX , Wen YX , et al. Apatinib combined with local irradiation leads to systemic tumor control via reversal of immunosuppressive tumor microenvironment in lung cancer. Cancer Res Treat. 2020;52:406‐418. 31476848 10.4143/crt.2019.296 PMC7176950

[24] Siegel RL , Miller KD , Jemal A . Cancer statistics, 2020. CA Cancer J Clin. 2020;70:7‐30. 31912902 10.3322/caac.21590

[25] Chun SG , Hu C , Choy H , et al. Impact of intensity‐modulated radiation therapy technique for locally advanced non‐small‐cell lung cancer: a secondary analysis of the NRG oncology RTOG 0617 randomized clinical trial. J Clin Oncol. 2017;35:56‐62. 28034064 10.1200/JCO.2016.69.1378 PMC5455690

[26] Tian S , Quan H , Xie C , et al. YN968D1 is a novel and selective inhibitor of vascular endothelial growth factor receptor‐2 tyrosine kinase with potent activity in vitro and in vivo. Cancer Sci. 2011;102:1374‐1380. 21443688 10.1111/j.1349-7006.2011.01939.x PMC11158267

[27] Feng H , Cheng X , Kuang J , et al. Apatinib‐induced protective autophagy and apoptosis through the AKT‐mTOR pathway in anaplastic thyroid cancer. Cell Death Dis. 2018;9:1030. 30301881 10.1038/s41419-018-1054-3 PMC6177436

[28] Meng X , Wang H , Zhao J , et al. Apatinib inhibits cell proliferation and induces autophagy in human papillary thyroid carcinoma via the PI3K/Akt/mTOR signaling pathway. Front Oncol. 2020;10:217. 32219060 10.3389/fonc.2020.00217 PMC7078169

[29] Lu W , Ke H , Qianshan D , Zhen W , Guoan X , Honggang Y . Apatinib has anti‐tumor effects and induces autophagy in colon cancer cells. Iran J Basic Med Sci. 2017;20:990‐995. 29085592 10.22038/IJBMS.2017.9263 PMC5651465

[30] Cheng X , Feng H , Wu H , et al. Targeting autophagy enhances apatinib‐induced apoptosis via endoplasmic reticulum stress for human colorectal cancer. Cancer Lett. 2018;431:105‐114. 29859300 10.1016/j.canlet.2018.05.046

[31] Xie C , Zhou X , Liang C , et al. Apatinib triggers autophagic and apoptotic cell death via VEGFR2/STAT3/PD‐L1 and ROS/Nrf2/p62 signaling in lung cancer. J Exp Clin Cancer res. 2021;40:266. 34429133 10.1186/s13046-021-02069-4 PMC8385858

[32] Yu R , Bai H , Gao B , et al. Rare case of apatinib acquired resistance induced by point mutation of WRN p.V697F through activation of the PI3K/AKT apoptosis‐inhibiting pathway. Thorac Cancer. 2021;12:128‐132. 33225619 10.1111/1759-7714.13726 PMC7779201

[33] Wang YM , Xu X , Tang J , et al. Apatinib induces endoplasmic reticulum stress‐mediated apoptosis and autophagy and potentiates cell sensitivity to paclitaxel via the IRE‐1alpha‐AKT‐mTOR pathway in esophageal squamous cell carcinoma. Cell Biosci. 2021;11:124. 34229754 10.1186/s13578-021-00640-2 PMC8261945

[34] Song J , Guan Z , Song C , Li M , Gao Z , Zhao Y . Apatinib suppresses the migration, invasion and angiogenesis of hepatocellular carcinoma cells by blocking VEGF and PI3K/AKT signaling pathways. Mol Med Rep. 2021;23(6):429. 33846786 10.3892/mmr.2021.12068 PMC8047914

[35] Lan C , Shen J , Wang Y , et al. Camrelizumab plus Apatinib in patients with advanced cervical cancer (CLAP): a multicenter, open‐label, single‐arm, phase II trial. J Clin Oncol. 2020;38:4095‐4106. 33052760 10.1200/JCO.20.01920 PMC7768345

[36] Xu J , Shen J , Gu S , et al. Camrelizumab in combination with Apatinib in patients with advanced hepatocellular carcinoma (RESCUE): a nonrandomized, open‐label, phase II trial. Clin Cancer Res. 2021;27:1003‐1011. 33087333 10.1158/1078-0432.CCR-20-2571

[37] Zhao H , Yao W , Min X , et al. Apatinib plus gefitinib as first‐line treatment in advanced EGFR‐mutant NSCLC: the phase III ACTIVE study (CTONG1706). J Thorac Oncol. 2021;16:1533‐1546. 34033974 10.1016/j.jtho.2021.05.006

[38] Huang Y , Goel S , Duda DG , Fukumura D , Jain RK . Vascular normalization as an emerging strategy to enhance cancer immunotherapy. Cancer res. 2013;73:2943‐2948. 23440426 10.1158/0008-5472.CAN-12-4354 PMC3655127

[39] Liu M , Li H , Wang X , Jing L , Jiang P , Li Y . Experimental study of the vascular normalization window for tumors treated with apatinib and the efficacy of sequential chemotherapy with apatinib in lung cancer‐bearing mice and patients. Cancer Med. 2020;9:2660‐2673. 32073228 10.1002/cam4.2923 PMC7163088

[40] Xu J , Zhang Y , Jia R , et al. Anti‐PD‐1 antibody SHR‐1210 combined with Apatinib for advanced hepatocellular carcinoma, gastric, or esophagogastric junction cancer: an open‐label, dose escalation and expansion study. Clin Cancer res. 2019;25:515‐523. 30348638 10.1158/1078-0432.CCR-18-2484

[41] Zhu H , Yang X , Ding Y , et al. Recombinant human endostatin enhances the radioresponse in esophageal squamous cell carcinoma by normalizing tumor vasculature and reducing hypoxia. Sci Rep. 2015;5:14503. 26412785 10.1038/srep14503 PMC4585975

[42] Ciric E , Sersa G . Radiotherapy in combination with vascular‐targeted therapies. Radiol Oncol. 2010;44:67‐78. 22933894 10.2478/v10019-010-0025-9 PMC3423684

[43] Granton PV , Dubois L , van Elmpt W , et al. A longitudinal evaluation of partial lung irradiation in mice by using a dedicated image‐guided small animal irradiator. Int J Radiat Oncol Biol Phys. 2014;90:696‐704. 25200196 10.1016/j.ijrobp.2014.07.004

